# A bovine 3D endometrium-on-a-chip reveals the role of conceptus-derived proteins, CAPG and PDI, in conceptus–endometrial communication

**DOI:** 10.1093/biolre/ioaf077

**Published:** 2025-04-06

**Authors:** Haidee Tinning, Elton J R Vasconcelos, Dapeng Wang, Niamh Forde

**Affiliations:** Discovery and Translational Sciences Department, Leeds Institute of Cardiovascular and Metabolic Medicine, Faculty of Medicine and Health, University of Leeds, Leeds, West Yorkshire, United Kingdom; LeedsOmics, University of Leeds, Leeds, West Yorkshire, United Kingdom; LeedsOmics, University of Leeds, Leeds, West Yorkshire, United Kingdom; National Heart and Lung Institute, Imperial College London, London, United Kingdom; Discovery and Translational Sciences Department, Leeds Institute of Cardiovascular and Metabolic Medicine, Faculty of Medicine and Health, University of Leeds, Leeds, West Yorkshire, United Kingdom

**Keywords:** microfluidics, proteomics, transcriptomics, uterine luminal fluid, periimplantation, receptivity

## Abstract

Early embryo loss affects all mammalian species, including humans, and agriculturally important food-producing mammals such as cattle. The developing conceptus (embryo and extraembryonic membranes) secretes proteins that can modify the endometrium and can be critical for early pregnancy processes, such as maternal recognition of pregnancy (MRP) or enhancing uterine receptivity to implantation. For example, a competent bovine conceptus secretes interferon tau (IFNT) to initiate MRP. The bovine conceptus also secretes other proteins at the time of MRP, including CAPG and PDI, which are highly conserved among placental mammals. We have previously shown that these proteins act upon the endometrium to modulate receptivity, embryo development, and implantation in species with different implantation strategies (humans and cattle). We hypothesize that developing a novel 3D bovine endometrium-on-a-chip system will enhance our understanding of the role of conceptus-derived factors in altering the endometrium and/or uterine luminal fluid (ULF) secretion. Here, we have developed a 3D bovine endometrium-on-a-chip system, comprising both stromal and epithelial cell culture combined with culture medium flow. This system better mimics the in vivo endometrium, and endometrial exposure to conceptus-derived factors, than conventional 2D endometrial cell culture*.* We have demonstrated that the conceptus-derived proteins, CAPG and PDI, modulate the endometrial transcriptome and secretory response to promote pathways associated with early pregnancy and alter ULF composition. This work highlights the critical need for more robust and in vivo-like culture systems to study endometrial–conceptus interactions in vitro to further investigate the role of conceptus-derived factors for pregnancy success.

## Teaser

Our 3D bovine endometrium-on-a-chip better recapitulates the endometrium in vitro to reveal the role of the conceptus-derived proteins, CAPG and PDI, in promoting changes to the endometrial transcriptome and secretome.

## Graphical Abstract

**Graphical Abstract:**
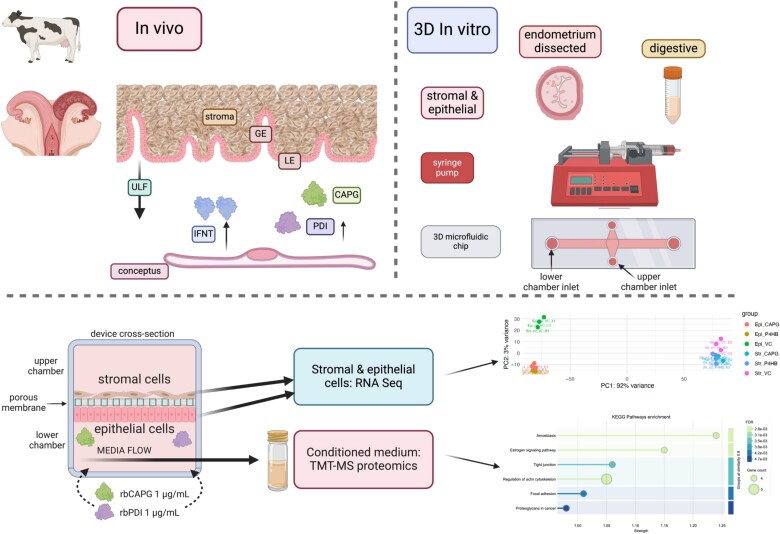

## Introduction

The endometrium, the inner lining of the uterine cavity and a highly heterogeneous specialized tissue, is primarily composed of epithelial and stromal cell types, but also contains microvasculature, immune cells, and stem cells identified in some species [1]. The epithelial cells form a complete monolayer lining the internal cavity of the uterus and can be subcategorized into luminal or glandular epithelial cells. Stromal cells lie below the epithelial monolayer, making up the bulk of the endometrial tissue [2].

The epithelial cells, and particularly the glandular epithelial cells (in the form of uterine glands), secrete histotroph into the uterine cavity, which contributes to the uterine luminal fluid (ULF) found within the uterine cavity. The ULF can also contain secretions from the oviductal cells [3] and from an embryo/conceptus itself if present [4, 5]. The ULF is the source of nutrition for the developing embryo/conceptus prior to placentation [6]. Studies involving endometrial epithelial gland knockouts in both mouse and sheep have demonstrated the critical importance of endometrial glands supporting early pregnancy, particularly conceptus elongation and the implantation process [7–9].

Much of what is known about early pregnancy in mammals has been achieved using in vivo animal studies or traditional in vitro techniques, which involve the culture of cells (either primary or immortalized). In vivo studies require large numbers of animals to be sufficiently powerful to produce statistically significant results, are extremely expensive, often require ethical approval, and require a lot of hands-on work and sample processing [10]. In vivo techniques may also not be able to identify low-abundant molecules (such as conceptus-derived factors) due to these being diluted by bodily fluids (such as ULF). Although invaluable, in vivo animal work can be difficult to achieve and to interpret the results due to variability between animals and between study designs [10]. In addition, initiatives such as NC3Rs aim to reduce, replace, and refine the use of animals in research [11], and as such, alternatives to in vivo animal research should be explored at every opportunity.

As an alternative to in vivo models*,* in vitro cell culture studies are well established and routinely used in many laboratories, can be high throughput, and do not usually require ethical approval. However, it has been demonstrated that cells grown in this manner experience abnormal proliferation and differentiation [12]. Cell culture growth medium is usually added to a culture vessel and left static for a period (often 48 h or more), resulting in depleted nutrient availability and increased metabolic waste exposure [13], neither of which represents most biological systems. Additionally, conventional cell culture is conducted on a 2D flat culture ware surface, with adherent cells growing in a monolayer [12]. This does not well recapitulate most biological tissues or systems, including the endometrium. Tissues are more complex containing multiple cell types in a three-dimensional (3D) conformation, which contributes to their function. Innovative approaches to the in vitro culture of mammalian cells aim to overcome some of these limitations and bridge the gap between in vivo animal studies and in vitro traditional culture techniques [12, 14].

Advances in 3D modeling approaches have identified and produced in vitro models that better recapitulate the in vivo tissue structure or environment [14]. Recent examples include microfluidic systems [15], organ-on-a-chip devices [16], scaffolds/extracellular matrix culture supports [17], and porous membranes [18]. These novel techniques have been developed to overcome the limitations of conventional in vitro cell culture and can be applied to investigating endometrial–conceptus interactions. Microfluidic systems provide the opportunity to produce systems where fluid can be pushed through a small chip/channel at a set rate of flow [15]. The channel must be less than 1 mm in width or depth to be classified as a microfluidics device [19]. Initially designed to study the physics of fluid movement, microfluidics can be combined with cell culture by loading cells into the chip or channel before applying flow and using culture medium as fluid within the system. Microfluidics can be used to recapitulate a flow rate similar to that of the system being investigated; for this reason, microfluidics is often used in the study of blood vessel formation and function under high shear stress [20]. Culturing cells in medium that is continuously replenished better mimics the in vivo environment, as in vivo the cells are continuously nutritionally supplied and metabolic waste is removed by the circulatory system*.* By using a device designed to culture multiple cell types, usually in a 3D structure or in series (i.e., different cell types in a series of compartments), it is possible to recapitulate a certain organ or system [16]. Organ-on-chip (OoC) approaches have been developed for many aspects of reproduction, including the oviduct [21], placenta [22], and the process of implantation [23], and were recently reviewed in detail [24]. Many groups have fabricated endometrial microfluidic systems in humans, including a 3D system incorporating endometrial epithelial, stromal, and microvasculature cells under flow [25]. A more complex example of endometrium bioengineering is the development of a multiorgan-on-a-chip system, which recapitulates the human menstrual cycle through a series of compartments containing “on-a-chip” versions of the ovary, fallopian tube, uterus, cervix, and liver [26]. The system was shown to develop follicles in vitro, secrete steroid hormones, and tissues maintained their in vivo-like structures in vitro [26]*.* Specifically, in bovine, a recent study described a system whereby bovine endometrial stromal and epithelial cells could be co-cultured, with stromal cells exposed to varying concentrations of glucose and insulin, to mimic the endometrial exposure to factors in the maternal circulation [27].

We therefore aimed to use a 3D organ-on-a-chip bovine endometrium in combination with microfluidics, to study the conceptus–endometrial communication which occurs in vivo around the critical period of MRP. Specifically, we tested the hypothesis that a 3D cell culture microfluidics approach will allow us to understand the functional roles of conceptus-derived proteins during pregnancy recognition. To test this hypothesis, we aimed to 1) develop an in vitro 3D bovine endometrial model in a microfluidic system comprising both epithelial and stromal endometrial cells, 2) identify the secretome of the on-a-chip bovine endometrium to compare to in vivo ULF, and 3) utilize the endometrium-on-a-chip to investigate how the addition of the conceptus-derived proteins impacts the endometrial transcriptome and secretome.

## Materials & methods

Unless otherwise stated, all materials were purchased from Sigma-Aldrich.

### Endometrium-on-a-chip

#### Primary endometrial cell isolation

Bovine endometrial epithelial cells (bEECs) and bovine endometrial stromal cells (bESCs) were isolated from uterine tracts obtained from the local abattoir as described in detail in Ref. [28]. Three uterine tracts in the late-luteal stage of the estrous cycle were selected based on the morphology of the ovaries to represent the appropriate stage of the cycle where the endometrium is receptive and would be exposed to conceptus-derived factors [29]. bESCs and bEECs were cultured in complete bovine medium (RPMI 1640, 10% dextran-coated charcoal-stripped FBS [PAA Cell Culture Company], 1% ABAM) in standard cell culture flasks and purified for 13–14 days postisolation to generate epithelial-enriched and stromal-enriched cell populations. During this time, the bESCs were passaged at a 1:3 ratio on day 7. Differential trypsinization was used to purify the cells, as stromal cells lift from the flask within 1–3 min when exposed to 0.025% trypsin, whereas epithelial cells require 5–10 min exposure to 0.25% trypsin. Cells were then visually assessed for purity using a light microscope to be over 95% enriched for either bESCs or bEECs, as previously confirmed by flow cytometry [27].

#### Seeding of endometrium-on-a-chip device

Cells were trypsinized, washed in phosphate-buffered saline (PBS), and resuspended in complete bovine medium with 10% exosome-depleted FBS (Gibco); bESCs (passage 2) were adjusted to 200,000 cells/mL, and bEECs (passage 1) were adjusted to 1,000,000 cells/mL. Fifty-five μL (11,000 cells) of the bESC solution was added to the upper static chamber of a μ-Slide Membrane ibiPore Flow ibiTreat microfluidic device (Ibidi, 0.5-μm porous glass membrane, 20% porosity) and incubated for 1 h (38.5°C/5% CO_2_) to facilitate cell adherence (bESCs adhered to the top of the membrane). Two hundred μL bEEC solution (200,000 cells) was then added to the lower chamber. All caps were added to the inlets/outlets to prevent evaporation, immediately inverted (so bEECs adhered to the underside of the membrane), placed into a sterile petri dish, and incubated (38.5°C/5% CO_2_) overnight. Each device was seeded with epithelial and stromal cells isolated from the same animal. No additional coatings were used to promote cell adherence to the device. The microfluidic device was then de-inverted, the lower chamber caps and medium removed, and replenished with exosome-depleted bovine medium (detailed above). The chip was then returned to the incubator (38.5°C/5% CO_2_) for 3–4 days and the bEEC layer confluency visually determined. Medium was replenished every two days from the lower channel inlet.

#### Microfluidic flow treatment system

**Figure 1:**
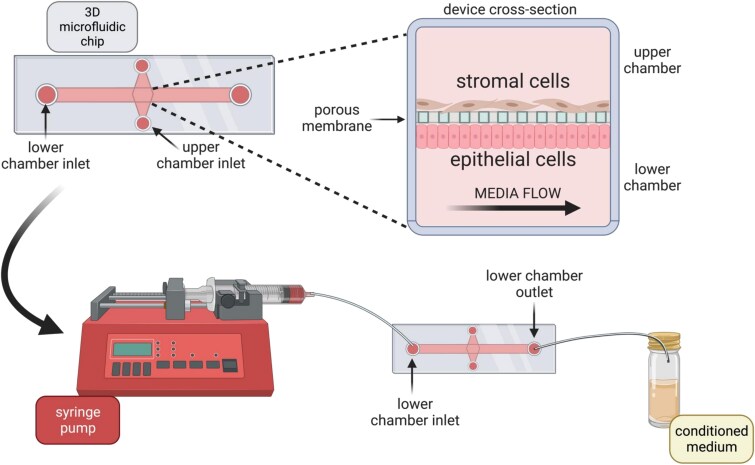
*Design of 3D bovine endometrial Ibidi organ-on-a-chip microfluidic system.* Lower chamber has an inlet on the left, and outlet on the right, which are connected via adaptors and tubing to a syringe containing medium. The syringe is connected to a syringe pump which pushes the syringe plunger to push medium through the lower chamber at 0.8 μL per min. Conditioned medium is collected from the lower chamber outlet. Upper chamber is separated by a porous glass membrane and is static (not under flow). bEECs seeded in lower chamber to underside of membrane and bESCs seeded in upper chamber to confluency. Figure created using biorender.com.

Recombinant bovine forms of CAPG (rbCAPG) and PDI (rbPDI) were produced as described [28, 30] by Newcastle University Protein and Proteome Analysis Facility (UK) and purified into PBS. Treatments were prepared in exosome-depleted complete bovine medium as follows: 1) vehicle control (VC-PBS), 2) rbCAPG 1000 ng/mL, or 3) rbPDI 1000 ng/mL. Concentrations were previously shown to alter the transcriptome in 2D static culture systems [28, 30]. Treatments were loaded into 5-mL Luer lock syringes (Terumo), capped, and pre-warmed in the incubator to 38.5°C overnight. Bubbles were then removed from the syringes by tapping. A shelf was removed from the incubator, cleaned with 70% ethanol, and placed into a sterile laminar flow hood. A syringe pump (New Era Pump Systems) was wiped with 70% ethanol and placed into the hood on the incubator shelf. The pre-warmed syringes were secured in the pump system, and 0.8-mm ID sterile silicone tubing (Ibidi) was attached with a Luer lock female connector (Ibidi) to the syringe. The tubing was then attached to an elbow Luer connector (Ibidi) and the syringes were manually pushed to fill the tubing with medium until a droplet appeared at the outlet of the elbow Luer connector. The devices were removed from the incubator, and the lower channel was flushed with PBS three times by adding to the inlet and removing from the outlet. The lower channel inlet and outlet were then overfilled to produce a dome of liquid at the surface. The droplet leaving the tubing from the syringe was then connected to the domed liquid inside the lower channel. This linking process was necessary to prevent bubbles. The elbow Luer connector male was then pushed at a 90-degree angle into lower channel inlet of the device and turned back 90 degrees to seal. A short piece of sterile tubing was pre-prepared with a second elbow Luer connector male attached to one end. On the other end, a 1-mL syringe (BD Plastipak) with a blunt needle (SOL-Millennium) was inserted and used to push PBS into the tubing until a droplet formed from the outlet of the elbow Luer connector male. This droplet was then linked to that of the device’s domed medium of the lower channel outlet, again to prevent bubbles from being trapped inside the microfluidic system. The needle was simultaneously removed from the outlet tubing whilst pushing the outlet elbow Luer connector male into the chip outlet at a 90-degree angle and sealing by twisting back 90 degrees. The syringes were then very gently pushed slowly to inject medium through the chip and microfluidic tubing system until the whole system was filled with medium. The end of the outlet tubing was then pushed into a hole made in the lid of a 7-mL sterile bijou to collect the conditioned medium during flow. The complete flow system on the shelf was transferred back to the incubator (38.5°C/5% CO_2_). The pump device was then plugged in, and flow rate was set to 0.8 μL/min and set to run for 24 h to mimic what is produced in vivo, as described below. Two 1 mL samples of VC medium were also placed in the incubator alongside the system for 24 h in duplicate (unconditioned medium). A graphical representation of the 3D endometrium-on-a-chip is seen in Figure 1.

The flow rate of 0.8 μL/min was determined for the 3D endometrium microfluidic channel based on previous experiments [27, 30]. An initial 1 μL/min flow rate was chosen because uterine tubal secretions were found to be 1.43 mL/24 h during diestrus and 1.54 mL/24 h during estrus in cattle [31], with the rate of 1 μL/min equating to 1.44 mL/24 h. To attempt to match the shear stress experienced by the epithelial cells under flow, the flow rate of 1 μL/min was changed to 0.8 μL/min for the 3D culture system based on the dimensions of the channel.

#### Sample collection

After 24 h, the flow of medium was stopped, the pump unplugged, and the whole system moved back to the laminar flow hood. First, the conditioned medium and unconditioned samples were transferred to 2-mL sterile microfuge tubes and stored at 4°C until processing. Then, the syringes were detached from the tubing, and all connectors were uncoupled from the devices and the tubing separated. The devices were flushed with PBS, and the lower flow channel was flushed three times. The upper channel was flushed with 55 μL PBS by using two P200 pipettes, one to push in 55 μL PBS to the inlet and the other to simultaneously extract 55 μL liquid from the outlet—this was also repeated three times. At this point, the devices were visually inspected under a light microscope to ensure the membrane was intact before continuing. Both the upper and lower channels were filled with trypsin solution (0.025% for bESCs or 0.25% for bEECs) and incubated for 3 min. Exosome-depleted complete bovine medium was added to each channel, and cells were collected from the outlets. Device was checked via light microscopy for any remaining adhered cells and to ensure the membrane was still intact, and the trypsinization processes were repeated if needed. Collected cells were centrifuged to pellet at 500 g for 5 min, resuspended in 1 mL PBS to wash, and re-pelleted at 500 g for 5 min. Supernatant was aspirated gently and cell pellets were snap-frozen in liquid nitrogen and transferred to a −80°C freezer.

The conditioned medium collected from the microfluidic lower-chamber flow-through and the unconditioned medium samples were processed to remove debris. First, the medium was centrifuged at 500 g for 10 min to pellet cells. Supernatant was carefully transferred to a new microfuge tube, and the pellet discarded. Then, the supernatant was subjected to a 2000 g 10 min spin to pellet cell debris. Supernatant was carefully transferred to a new Eppendorf, and the pellet discarded. Finally, the supernatant was centrifuged at 14,000 g for 30 min to pellet microvesicles. The resulting supernatant was snap-frozen and stored at −80°C.

### Transcriptome analysis

RNA was extracted from both the bEEC and bESC pellets using the miRNeasy Micro Kit (Qiagen) as per manufacturer’s instructions with on-column DNAse treatment (Qiagen), snap-frozen in liquid nitrogen, and stored at −80°C. RNA libraries were prepared and sequenced by Novogene (Cambridge) as per their standard protocols, which are described here based on information provided. Due to the small amount of RNA recovered, a SMARTer amplification process was performed using SMART-Seq v4 Ultra Low Input RNA Kit for sequencing (Clontech) to synthesize the double-stranded cDNA libraries. Briefly, first-strand cDNA synthesis was performed, followed by template switching and extension, then cDNA amplified by PCR to produce double-stranded cDNA. The amplified cDNA samples were purified with AMPure XP beads and quantified with a Qubit 2.0 fluorometer (Life Technologies). Library prep was then carried out using the Novogene NGS RNA library prep set (PT042). Briefly, the cDNA samples were sheared by the Covaris system, then sheared fragments underwent end-repair, A-tailed, and ligated to sequencing adaptors. During this process, a 200 bp size selection was used. The resulting libraries were then checked with Qubit 2.0 fluorometer (Life Technologies), diluted to 2 ng/μL, insert size checked on an Agilent 2100 Bioanalyzer, and quantified to greater accuracy by qPCR. Quantified libraries were then pooled and sequenced using the Illumina NovaSeq 6000 machine (Illumina, California, USA) with a paired-end 150-bp length read.

RNA paired-end sequencing quality control was assessed through FastQC (www.bioinformatics.babraham.ac.uk/projects/fastqc) and MultiQC [32]. Both adapters and low-quality bases (QV < 20) were trimmed from extremities of reads using Trimmomatic [33] with a minimum read length of 30 bp. An average of 53.6 million reads per sample was kept post-trimming for downstream analyses. All libraries were mapped against the bovine ARS-UCD1.3 release-114 reference genome (retrieved from https://ftp.ensembl.org/pub/release-113/fasta/bos_taurus.ARS-UCD1.3.dna.toplevel.fa.gz) through STAR aligner [34] with default parameters. STAR-generated sorted BAM output files were used for assigning read counts to gene features with featureCounts [35] with the following parameters: -p -B -C -M -O --fraction. We relied on Bos_taurus.ARS-UCD1.3.113.gtf annotation file downloaded from https://ftp.ensembl.org/pub/release-113/gtf/bos_taurus.ARS-UCD1.3.113.gtf.gz.

FeatureCounts-generated read count matrix was used as input for differential expression (DE) analyses relying on the DeSeq2 negative binomial distribution model through a local fitting type and a 0.05 false discovery rate (FDR) threshold [36]. Several pairwise comparisons were performed setting the following as the fold-change ratio to be assessed: (i) “Epi_CAPG” / “Epi_VC”, (ii) “Epi_PDI” / “Epi_VC”, (iii) “Str_CAPG” / “Str_VC”, (iv) “Str_PDI” / “Str_VC”. The same read count table for all samples was also submitted to a principal component analysis (PCA) using the varianceStabilizingTransformation(dds, blind = TRUE) and plotPCA() functions from DESeq2 R package. All tools described in this paragraph were run under the R environment version 4.1.0.

Only protein-coding genes and lncRNAs were retained for further analysis. Overrepresentation enrichment analysis of differentially expressed protein-coding gene sets was executed using STRING DB (string-db.org) [37] using the default parameters (FDR < 0.05). Venn diagram analysis was performed using Venny 2.1.0 (bioinfogp.cnb.csic.es) [38]. The data discussed in this publication have been deposited in NCBI’s Gene Expression Omnibus [39] and are accessible through GEO Series accession number GSE287563.

### Proteomics analysis of conditioned medium

#### TMT labeling and high-pH reversed-phase chromatography

The samples were depleted of bovine albumin using an albumin depletion kit, according to the manufacturer’s protocol (Thermo Fisher Scientific, Loughborough, LE11 5RG, UK). An equal volume of each depleted sample (equivalent to 20–50 μg protein) was then digested with trypsin (1.25 μg trypsin; 37°C, overnight), labeled with tandem mass tag (TMT) 11 plex reagents according to the manufacturer’s protocol (Thermo Fisher Scientific) and the labeled samples pooled.

The pooled sample was desalted using a SepPak cartridge according to the manufacturer’s instructions (Waters, Milford, Massachusetts, USA). Eluate from the SepPak cartridge was evaporated to dryness and resuspended in buffer A (20 mM ammonium hydroxide, pH 10) prior to fractionation by high-pH reversed-phase chromatography using an Ultimate 3000 liquid chromatography system (Thermo Fisher Scientific). In brief, the sample was loaded onto an XBridge BEH C18 Column (130 Å, 3.5 μm, 2.1 mm × 150 mm, Waters, UK) in buffer A and peptides eluted with an increasing gradient of buffer B (20 mM Ammonium hydroxide in acetonitrile, pH 10) from 0 to 95% over 60 min. The resulting fractions (15 in total) were evaporated to dryness and resuspended in 1% formic acid prior to analysis by nano-LC MS/MS using an Orbitrap Fusion Lumos mass spectrometer (Thermo Scientific).

#### Nano-LC mass spectrometry

High-pH RP fractions were further fractionated using an Ultimate 3000 nano-LC system in line with an Orbitrap Fusion Lumos mass spectrometer (Thermo Scientific). In brief, peptides in 1% (vol/vol) formic acid were injected onto an Acclaim PepMap C18 nano-trap column (Thermo Scientific). After washing with 0.5% (vol/vol) acetonitrile 0.1% (vol/vol) formic acid peptides were resolved on a 250 mm × 75 μm Acclaim PepMap C18 reverse-phase analytical column (Thermo Scientific) over a 150-min organic gradient, using seven gradient segments (1–6% solvent B over 1 min, 6–15% B over 58 min, 15–32% B over 58 min, 32–40% B over 5 min, 40–90% B over 1 min, held at 90% B for 6 min and then reduced to 1% B over 1 min.) with a flow rate of 300 nL/min. Solvent A was 0.1% formic acid, and Solvent B was aqueous 80% acetonitrile in 0.1% formic acid. Peptides were ionized by nano-electrospray ionization at 2.0 kV using a stainless steel emitter with an internal diameter of 30 μm (Thermo Scientific) and a capillary temperature of 300°C.

All spectra were acquired using an Orbitrap Fusion Lumos mass spectrometer controlled by Xcalibur 3.0 software (Thermo Scientific) and operated in data-dependent acquisition mode using an SPS-MS3 workflow. FTMS1 spectra were collected at a resolution of 120,000, with an automatic gain control (AGC) target of 200 000 and a max injection time of 50 ms. Precursors were filtered with an intensity threshold of 5000, according to charge state (to include charge states 2–7) and with monoisotopic peak determination set to peptide. Previously interrogated precursors were excluded using a dynamic window (60 s +/−10 ppm). The MS2 precursors were isolated with a quadrupole isolation window of 0.7 m/z. ITMS2 spectra were collected with an AGC target of 10 000, max injection time of 70 ms, and CID collision energy of 35%.

For FTMS3 analysis, the Orbitrap was operated at 50 000 resolution with an AGC target of 50 000 and a max injection time of 105 ms. Precursors were fragmented by high-energy collision dissociation (HCD) at a normalized collision energy of 60% to ensure maximal TMT reporter ion yield. Synchronous precursor selection (SPS) was enabled to include up to 10 MS2 fragment ions in the FTMS3 scan.

#### Data analysis

The raw data files were processed and quantified using Proteome Discoverer software v2.4 (Thermo Scientific) and searched against the UniProt *Bos taurus* database (downloaded October 2024: 59 258 entries) using the SEQUEST HT algorithm. Peptide precursor mass tolerance was set at 10 ppm, and MS/MS tolerance was set at 0.6 Da. Search criteria included oxidation of methionine (+15.995 Da), acetylation of the protein N-terminus (+42.011 Da), methionine loss from the N-terminus (−131.04 Da) and methionine loss plus acetylation of the protein N-terminus (−89.03 Da) as variable modifications and carbamidomethylation of cysteine (+57.0214) and the addition of the TMT mass tag (+229.163) to peptide N-termini and lysine as fixed modifications. Searches were performed with full tryptic digestion and a maximum of two missed cleavages was allowed. The reverse database search option was enabled, and all data were filtered to satisfy an FDR of 5%. The mass spectrometry proteomics data have been deposited in the ProteomeXchange Consortium via the PRIDE [40] partner repository with the dataset identifier PXD059725.

The resulting list of proteins for each sample was then analyzed in Microsoft Excel for Mac (version 16.92) with Real Statistics Resource Pack software (release 8.9.1, www.real-statistics.com) to determine the fold change in protein abundance between treatment groups and the associated p-value [41]*.* Briefly, as described in detail by Aguilan et al. [41], the data were first filtered to remove any proteins that had no quantitative values (abundance column in Supplementary Table S1) in any sample or replicate (COUNT column in Supplementary Table S1). Then, the data were log2 transformed, normalized by average, and then normalized by slope. Imputation was then performed to replace missing values using the probabilistic minimum imputation for label-free data method, replacing missing values with those of a normal distribution. Fold changes were calculated by taking the average of the vehicle control samples from the average of the rbPDI-/rbCAPG-treated conditioned medium samples. Fold changes for the “in vitro ULF” were calculated by taking the average of the unconditioned medium samples from the average of the vehicle control samples. Proteins with a positive fold change are therefore more abundant, and those with a negative fold change value are less abundant. For rbCAPG/rbPDI samples, p-value significance was calculated by a paired two-tailed *t*-test. All comparisons with a p-value <0.05 were considered significantly different. For the in vitro ULF samples, first, an *F*-test was carried out to determine which proteins displayed a different variance between conditioned and unconditioned samples, as the samples were not paired. For the proteins which had an *F*-test value p < 0.05 a two-tailed two-sample unequal variance t-test was performed, and those that had an *F*-test value p > 0.05 a two-tailed two-sample equal variance *t*-test was performed. Full data shown in Supplementary Table S1. Protein lists were then filtered for p < 0.05 and excluded any that were identified as false for bovine and/or true for contaminant. All significantly differentially abundant proteins between rbCAPG/rbPDI vs vehicle controls were present in all replicates in all treatment groups. All but one of the significantly differentially abundant proteins were present in all samples in the unconditioned vs vehicle control samples. The protein signal peptidase complex subunit 2 (E1BEI2) was only found in one replicate of the unconditioned medium samples. The count of samples with positive abundance per protein and raw abundance data can be seen in Supplementary Table S1.

#### Proteomics data downstream analysis

To first investigate the data, the list of proteins and imputation values (post-normalization, shown in Supplementary Table S1, were placed into a .txt table and loaded into RStudio version 4.2.2 [42]. A box plot was produced using BioStatR version 4.0.1 [43]. A PCA plot was produced using ggfortify version 0.4.16 [44]. Then, to visualize the in vitro ULF data, a volcano plot was produced using the EnhancedVolcano Bioconductor package version 1.16.0 ([45]). This list of in vitro produced ULF proteins identified was then compared to those identified in vivo in other studies, including Forde et al. [5]*.* Data by Forde *et al* were taken from their Supplemental Table S1, which identified 334 proteins present in the ULF from at least three out of four day 16 non-pregnant cattle. The list of 334 proteins was identified by a “NCBI GI number” and so was converted to UniProtKB/Swiss-Prot identifiers to match the identifiers used in this study. One hundred and forty-three proteins were converted to bovine UniProt KB Swiss Prot identifiers using the UniProt ID mapping online tool (uniprot.org/id-mapping) [46]. Enrichment and protein–protein association analysis were carried out using STRING DB (string-db.org) [47].

## Results

### 3D bovine endometrium-on-a-chip secretome

**Figure 2:**
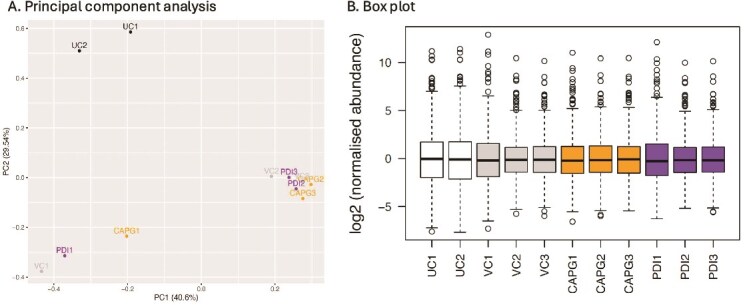
*Overall proteomic analysis of conditioned medium in 3D endometrium-on-a-chip. A.* Principal component analysis plot of conditioned medium from 3D microfluidics device (in vitro ULF). *B.* Boxplot of normalized abundance values for proteins identified in the in vitro ULF. Conditioned medium flowed through bEEC culture chamber connected via porous membrane to static bESC culture chamber. Treatments were added to culture medium flowed through the bEEC chamber at a rate of 0.8 μL/min for 24 h and contained one of the following treatments in biological triplicate: VC = vehicle control, PDI = rbPDI 1 μg/mL, CAPG = rbCAPG 1 μg/mL. Numbers following VC, PDI, or CAPG samples indicate biological replicate. Two unconditioned medium samples were also analyzed. Proteins identified in medium using TMT mass spectrophotometry. PCA and boxplot produced using ggplot in RStudio. Full data in Supplementary Table S1.

**Figure 3:**
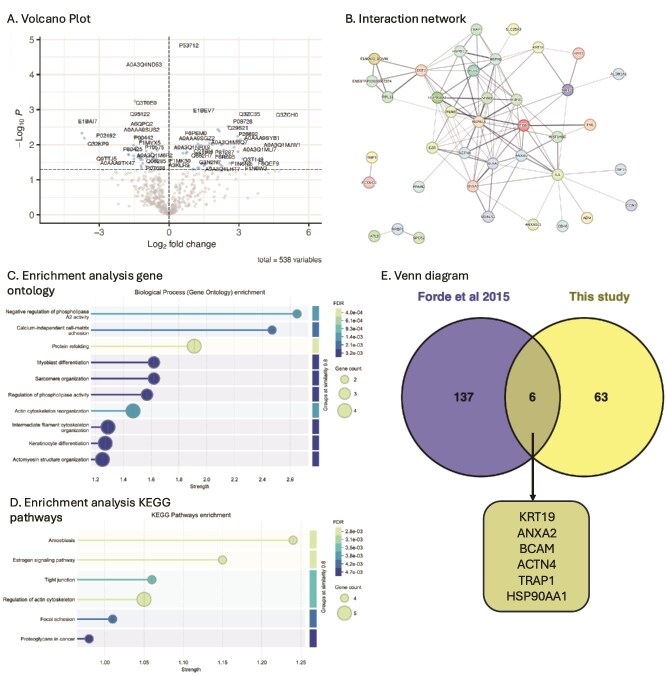
*Protein composition of in vitro endometrium-on-a-chip produced ULF. A.* Volcano plot of differentially abundant proteins presents in conditioned medium (vehicle control) samples compared to the unconditioned medium samples. Samples above the dashed line are significantly changed in abundance between the conditioned (n = 3) vs unconditioned samples (n = 2) (p < 0.05). Conditioned medium flowed through bEEC culture chamber at a rate of 0.8 μL/min for 24 h separated by a porous membrane to a static bESC culture chamber. Figure produced using EnhancedVolcano in R Studio. *B.* String DB analysis of in vitro ULF proteins. Each node represents a protein, and edges (connections) represent functional/physical protein associations with a minimum required interaction score of “medium 0.4”. Thickness of edge represents the strength of supporting data. In vitro secreted proteins determined by comparing conditioned medium produced by the endometrium-on-a-chip system (n = 3 vehicle controls) flowed through the bEEC chamber connected to a static bESC chamber via a porous glass membrane, to the unconditioned medium (n = 2) samples (p < 0.05). Produced using STRING DB. Disconnected nodes removed for clarity *C.* Enriched GO terms and *D.* enriched KEGG pathways associated with proteins secreted in the in vitro ULF. Proteins significantly enriched (positive fold change abundance) in conditioned vehicle control medium samples (n = 3) compared to unconditioned samples (n = 2) (p < 0.05), from endometrium-on-a-chip culture system, were subjected to go term enrichment analysis in STRING DB (FDR <0.05). Strength of enrichment is Log10(observed/expected). Full data in Supplementary Tables S3 and S4. *E.* Venn diagram comparing in vivo ULF proteins to in vitro secreted ULF proteins. In vivo ULF proteins identified by Forde et al. [5] as present in day 16 non-pregnant cattle compared to in vitro ULF proteins identified as secreted by the 3D endometrium-on-a-chip microfluidic system described here. ULF = uterine luminal fluid. Full data in Supplementary Table S5.

Following proteomics analysis of the conditioned medium, PCA was used to visualize the spread of the data after normalization. The PCA plot (Figure 2A) showed the conditioned medium samples clustered mostly by biological replicate (in PC1), with the two unconditioned medium samples clustered together at the top of the plot (in PC2). The data were then investigated to assess the spread of the individual data points using a box plot (Figure 2B), demonstrating that the data were similar across all sample types. To determine what in vitro ULF secretion occurred, proteins present in the conditioned vehicle control samples produced from the 3D endometrium-on-a-chip microfluidics system that were significantly increased or decreased (p < 0.05) in abundance when compared to the unconditioned medium samples were identified (Figure 3A; Supplementary Table S2). Those that were significantly increased in abundance were then identified (Table 1) and were collectively termed “in vitro ULF” as they were secreted into the conditioned medium in this endometrial model system. Of the 69 enriched in vitro ULF proteins, 49 were mapped by STRING DB. STRING DB interaction analysis of the 49 proteins demonstrated that many of the proteins secreted from the 3D endometrial chip are associated and/or interact with each other (i.e., have edges connecting them to other proteins) (Figure 3B). There were one large cluster and two small unconnected clusters of proteins: metalloendopeptidase (BMP1) and procollagen c-endopeptidase enhancer (PCOLCE), and ATLastin GTPase 3 (ATL3), ribosome binding protein (RRBP1), and signal peptidase complex subunit 2 (SPCS2) (names taken from STRING DB).

**Table 1:** List of “in vitro” ULF secreted proteins. Conditioned medium produced by the endometrium-on-a-chip system conditioned medium flowed through the bEEC chamber. bEEC chamber connected to a static bESC chamber via a porous glass membrane. Enriched proteins present in conditioned medium (n = 3, vehicle control) samples compared to the unconditioned medium (n = 2) samples (p < 0.05). Fold-change and t-test carried out in Excel as described by Aguilan et al. [41]*.* OS = OrganismName, OX = OrganismIdentifier, GN = GeneName, PE = ProteinExistence, SV = SequenceVersion

Accession	Description	*t*-test	fold change
Q3ZCH0	Stress-70 protein, mitochondrial OS=*Bos taurus* OX = 9913 GN=HSPA9 PE = 2 SV = 1	0.00139641	5.05757182
A0A3Q1MJW1	Heterogeneous nuclear ribonucleoproteins A2/B1 OS=*B. taurus* OX = 9913 GN=HNRNPA2B1 PE = 1 SV = 1	0.01010148	4.86060995
F6QEF9	High-mobility group protein HMG-I/HMG-Y OS=*B. taurus* OX = 9913 GN=HMGA1 PE = 3 SV = 1	0.03461813	4.26987059
A0AAA9SYB1	Nephronectin OS=*B. taurus* OX = 9913 GN=NPNT PE = 3 SV = 1	0.00646317	3.99536839
A0A3Q1MLI7	Pro-adrenomedullin OS=*B. taurus* OX = 9913 GN = ADM PE = 3 SV = 1	0.01363125	3.89094397
F1N0W3	Large ribosomal subunit protein uL22 OS=*B. taurus* OX = 9913 PE = 3 SV = 2	0.04747611	3.73549228
F6S1Q0	Keratin 18 OS=*B. taurus* OX = 9913 GN=KRT18 PE = 1 SV = 2	0.00595432	3.62102373
Q3T149	Heat shock protein beta-1 OS=*B. taurus* OX = 9913 GN=HSPB1 PE = 2 SV = 1	0.02644525	3.61720313
A0A3Q1MER0	Small ribosomal subunit protein uS10 domain-containing protein OS=*B. taurus* OX = 9913 PE = 3 SV = 1	0.03688554	3.55004792
Q3ZC35	CCN family member 1 OS=*B. taurus* OX = 9913 GN=CCN1 PE = 2 SV = 1	0.0013274	3.45643383
A0A3Q1LYU3	Atlastin GTPase 3 OS=*B. taurus* OX = 9913 GN = ATL3 PE = 3 SV = 2	0.03630029	3.44215146
P26892	Interleukin-6 OS=*B. taurus* OX = 9913 GN=IL6 PE = 2 SV = 1	0.00495612	3.39997164
P12234	Solute carrier family 25 member 3 OS=*B. taurus* OX = 9913 GN=SLC25A3 PE = 1 SV = 1	0.00128502	3.3199413
E1BEI2	Signal peptidase complex subunit 2 OS=*B. taurus* OX = 9913 GN=SPCS2 PE = 1 SV = 2	0.00677409	3.25501715
P08728	Keratin, type I cytoskeletal 19 OS=*B. taurus* OX = 9913 GN=KRT19 PE = 2 SV = 1	0.00218699	3.16550832
A0A3Q1MTA5	Annexin OS=*B. taurus* OX = 9913 GN = ANXA8L1 PE = 1 SV = 1	0.02515509	3.16184977
F1N6N2	G protein-coupled receptor class C group 5 member A OS=*B. taurus* OX = 9913 GN = GPRC5A PE = 4 SV = 2	0.03504473	3.09752545
F6R5A9	TNFR-Cys domain-containing protein OS=*B. taurus* OX = 9913 PE = 4 SV = 3	0.00726209	3.06538304
Q29S21	Keratin, type II cytoskeletal 7 OS=*B. taurus* OX = 9913 GN=KRT7 PE = 2 SV = 1	0.00330103	2.96285821
P48644	Aldehyde dehydrogenase 1A1 OS=*B. taurus* OX = 9913 GN = ALDH1A1 PE = 1 SV = 3	0.01577837	2.95551424
A0AAA9TRU9	Synaptotagmin binding cytoplasmic RNA interacting protein OS=*B. taurus* OX = 9913 GN=SYNCRIP PE = 1 SV = 1	0.00578826	2.94038927
A0A3Q1LJE5	Dynein axonemal intermediate chain 7 OS=*B. taurus* OX = 9913 GN=DNAI7 PE = 3 SV = 2	0.00754522	2.9313546
A0AAA9TLR8	Protein S100-A4 OS=*B. taurus* OX = 9913 GN=S100A4 PE = 3 SV = 1	0.01195811	2.79052541
A0AAA9T6F1	Reticulon OS=*B. taurus* OX = 9913 GN = RTN4 PE = 1 SV = 1	0.03394337	2.60423401
A0A3Q1M6Q7	Plectin OS=*B. taurus* OX = 9913 GN=PLEC PE = 1 SV = 2	0.00854117	2.589857
Q3SYU2	Elongation factor 2 OS=*B. taurus* OX = 9913 GN = EEF2 PE = 2 SV = 3	0.00300592	2.53961958
F6R695	WAP four-disulfide core domain protein 2 OS=*B. taurus* OX = 9913 GN=WFDC2 PE = 1 SV = 1	0.02242033	2.40262055
P81287	Annexin A5 OS=*B. taurus* OX = 9913 GN = ANXA5 PE = 1 SV = 3	0.01659094	2.38169653
P04272	Annexin A2 OS=*B. taurus* OX = 9913 GN = ANXA2 PE = 1 SV = 2	0.0143677	2.34661696
A0A3Q1LHT7	Ribosome binding protein 1 OS=*B. taurus* OX = 9913 GN = RRBP1 PE = 1 SV = 2	0.0498427	2.30072478
A0AAA9TCQ9	AHNAK nucleoprotein OS=*B. taurus* OX = 9913 GN = AHNAK PE = 1 SV = 1	0.04090394	2.27150044
P11116	Galectin-1 OS=*B. taurus* OX = 9913 GN = LGALS1 PE = 1 SV = 2	0.02270094	2.25462966
P46193	Annexin A1 OS=*B. taurus* OX = 9913 GN = ANXA1 PE = 1 SV = 2	0.01002995	2.24948081
Q2HJ74	Glycine amidinotransferase, mitochondrial OS=*B. taurus* OX = 9913 GN = GATM PE = 2 SV = 1	0.03719533	2.20664046
A0A3Q1MQ34	Filamin B OS=*B. taurus* OX = 9913 GN=FLNB PE = 1 SV = 1	0.00412659	2.16174031
A0A3Q1LVC7	Ezrin OS=*B. taurus* OX = 9913 GN = EZR PE = 1 SV = 1	0.00369976	2.09516894
A0AAA9TTT0	Tropomyosin 1 OS=*B. taurus* OX = 9913 GN = TPM1 PE = 1 SV = 1	0.03086807	2.084422
P68432	Histone H3.1 OS=*B. taurus* OX = 9913 PE = 1 SV = 2	0.01462282	2.06552462
Q27975	Heat shock 70-kDa protein 1A OS=*B. taurus* OX = 9913 GN=HSPA1A PE = 1 SV = 2	0.00994746	1.85779487
P31081	60-kDa heat shock protein, mitochondrial OS=*B. taurus* OX = 9913 GN=HSPD1 PE = 1 SV = 2	0.04442466	1.77972867
E1BCM3	Ring finger protein 215 OS=*B. taurus* OX = 9913 GN = RNF215 PE = 4 SV = 4	0.04414025	1.76151898
G3N2N7	Calpastatin OS=*B. taurus* OX = 9913 GN=CAST PE = 1 SV = 3	0.03134923	1.75560847
A0A3Q1NIF0	Suppressor of tumorigenicity 14 protein homolog OS=*B. taurus* OX = 9913 GN=ST14 PE = 3 SV = 1	0.00919116	1.73867926
A0AAF6Z0W1	Tetraspanin OS=*B. taurus* OX = 9913 GN=CD9 PE = 3 SV = 1	0.00699849	1.65298324
F1N415	Piccolo presynaptic cytomatrix protein OS=*B. taurus* OX = 9913 GN=PCLO PE = 4 SV = 4	0.01634554	1.63902882
Q76LV2	Heat shock protein HSP 90-alpha OS=*B. taurus* OX = 9913 GN=HSP90AA1 PE = 1 SV = 3	0.03573166	1.56556275
A5D7D1	Alpha-actinin-4 OS=*B. taurus* OX = 9913 GN = ACTN4 PE = 2 SV = 1	0.03587523	1.51627415
A0AAA9SH52	Tropomyosin 3 OS=*B. taurus* OX = 9913 GN = TPM3 PE = 1 SV = 1	0.0397162	1.49881124
E1BEV7	Metalloendopeptidase OS=*B. taurus* OX = 9913 GN=BMP1 PE = 4 SV = 3	0.00105104	1.4914068
Q2TBI4	Heat shock protein 75 kDa, mitochondrial OS=*B. taurus* OX = 9913 GN = TRAP1 PE = 2 SV = 1	0.01548942	1.47269384
F1MQ37	Myosin-9 OS=*B. taurus* OX = 9913 GN = MYH9 PE = 1 SV = 3	0.03375011	1.4089709
Q862H7	Protein S100 (Fragment) OS=*B. taurus* OX = 9913 GN=S100A11 PE = 1 SV = 1	0.02028692	1.40124894
E1BBL5	Growth differentiation factor 15 OS=*B. taurus* OX = 9913 GN = GDF15 PE = 3 SV = 3	0.0343462	1.38562079
A0A3Q1M3Z1	Membrane cofactor protein OS=*B. taurus* OX = 9913 GN=CD46 PE = 4 SV = 2	0.01058508	1.37344112
Q2HJB6	Procollagen C-endopeptidase enhancer OS=*B. taurus* OX = 9913 GN=PCOLCE PE = 2 SV = 1	0.04630534	1.29114066
A0AAA9SVD1	Protein disulfide-isomerase A3 OS=*B. taurus* OX = 9913 GN=PDIA3 PE = 3 SV = 1	0.00962281	1.24023743
A0AAA9STV6	Galectin OS=*B. taurus* OX = 9913 GN = LGALS3 PE = 1 SV = 1	0.04512895	1.22212586
A0AAA9SGZ2	Peroxidasin OS=*B. taurus* OX = 9913 GN=PXDN PE = 4 SV = 1	0.00650804	1.18787459
F6PSM0	Dickkopf WNT signaling pathway inhibitor 3 OS=*B. taurus* OX = 9913 GN=DKK3 PE = 3 SV = 1	0.00469896	1.14962269
Q9MZ08	Basal cell adhesion molecule OS=*B. taurus* OX = 9913 GN=BCAM PE = 2 SV = 2	0.01846971	1.14588341
A0AAA9SDR5	Elongation factor 1-alpha OS=*B. taurus* OX = 9913 PE = 3 SV = 1	0.04962782	1.03611334
A0A3Q1NHX9	Fibronectin OS=*B. taurus* OX = 9913 GN=FN1 PE = 4 SV = 1	0.0118817	0.97789813
A0A3Q1LKP0	Peroxisome proliferator activated receptor delta OS=*B. taurus* OX = 9913 GN=PPARD PE = 3 SV = 1	0.0424295	0.85865709
P53712	Integrin beta-1 OS=*B. taurus* OX = 9913 GN=ITGB1 PE = 1 SV = 3	1.4677E-05	0.84947772
A0AAA9SKR8	Ig-like domain-containing protein OS=*B. taurus* OX = 9913 PE = 4 SV = 1	0.01614933	0.7706866
A0AAA9TKA2	Chloride intracellular channel protein OS=*B. taurus* OX = 9913 GN=CLIC1 PE = 1 SV = 1	0.01760014	0.73214085
A0AAA9RXQ2	Pyruvate kinase OS=*B. taurus* OX = 9913 GN=PKM PE = 1 SV = 1	0.0175057	0.59218555
F1MK30	60S ribosomal protein L13 OS=*B. taurus* OX = 9913 GN = RPL13 PE = 3 SV = 2	0.0291477	0.46774254
A3KLR9	Superoxide dismutase [Cu-Zn] OS=*B. taurus* OX = 9913 GN=SOD3 PE = 1 SV = 1	0.03848034	0.42573493

Enrichment analysis revealed biological process gene ontology (GO) terms associated with proteins secreted in the in vitro ULF: negative regulation of phospholipase A2 activity, calcium-independent cell-matrix adhesion, and protein refolding as the most significantly enriched (Figure 3C: Supplementary Table S3). Enriched KEGG pathways include amebiasis, estrogen signaling pathway, regulation of actin cytoskeleton, and tight junction (Figure 3D, Supplementary Table S4).

To compare the in vitro ULF proteins secreted in the 3D endometrium-on-a-chip system to those produced in vivo*,* these data were compared to a key study that used mass spectrophotometry to identify proteins present in in vivo non-pregnant bovine ULF on day 16 [5]. This comparison determined that only 6 of the 69 proteins identified here were also found in in vivo ULF on day 16—keratin 19 (KRT19), annexin A2 (ANXA2), basal cell adhesion molecule (BCAM), alpha-actinin-4 (ACTN4), heat shock protein 75 (TRAP1), and heat shock protein HSP 90-alpha (HSP90AA1) (Figure 3E: Supplementary Table S5).

**Table 2:** List of differentially abundant proteins in culture medium following CAPG treatment. rbCAPG added to the culture medium flowing through the microfluidic bovine endometrium-on-a-chip device for 24 h and proteins present in the conditioned medium determined by mass spectrophotometry, differentially abundant proteins identified compared to vehicle control samples (n = 3 biological replicates). OS = OrganismName, OX = OrganismIdentifier, GN = GeneName, PE = ProteinExistence, SV = SequenceVersion

Accession	Description	*t*-test	fold change
A0AAF7A8X1	Capping actin protein, gelsolin like OS=*Bos taurus* OX = 9913 GN=CAPG PE = 1 SV = 1	0.00550567	3.91255064
A0AAA9SSR1	Ribonuclease inhibitor OS=*B. taurus* OX = 9913 GN = RNH1 PE = 1 SV = 1	0.04344961	0.86267244
A0A3Q1NHX9	Fibronectin OS=*B. taurus* OX = 9913 GN=FN1 PE = 4 SV = 1	0.0128835	0.85758831
P55859	Purine nucleoside phosphorylase OS=*B. taurus* OX = 9913 GN=PNP PE = 1 SV = 3	0.01924934	0.852828
A0A3Q1LYU3	Atlastin GTPase 3 OS=*B. taurus* OX = 9913 GN = ATL3 PE = 3 SV = 2	0.00373952	0.61717601
F1MDN4	60S acidic ribosomal protein P0 OS=*B. taurus* OX = 9913 GN = RPLP0 PE = 3 SV = 2	0.00994008	0.57278009
Q1RMR3	Cortactin OS=*B. taurus* OX = 9913 GN=CTTN PE = 1 SV = 1	0.04142314	0.4251496
P28801	Glutathione S-transferase P OS=*B. taurus* OX = 9913 GN = GSTP1 PE = 1 SV = 2	0.04601374	0.23242943
Q3SZ62	Phosphoglycerate mutase 1 OS=*B. taurus* OX = 9913 GN=PGAM1 PE = 2 SV = 3	0.03154839	0.11815717
F1MJK3	Pregnancy zone protein OS=*B. taurus* OX = 9913 GN = LOC506828 PE = 3 SV = 4	0.01650805	−0.0578733
Q3ZC35	CCN family member 1 OS=*B. taurus* OX = 9913 GN=CCN1 PE = 2 SV = 1	0.00177657	−0.1192773
E1BIP4	Elastin microfibril interfacer 2 OS=*B. taurus* OX = 9913 GN = EMILIN2 PE = 4 SV = 4	0.04494762	−0.1310066
A0AAA9TRZ3	receptor protein-tyrosine kinase OS=*B. taurus* OX = 9913 GN = EPHA4 PE = 4 SV = 1	0.0450689	−0.1539647
F1N0X0	Protocadherin 12 OS=*B. taurus* OX = 9913 GN=PCDH12 PE = 4 SV = 1	0.04256698	−0.2485719
Q5EA79	Galactose mutarotase OS=*B. taurus* OX = 9913 GN = GALM PE = 2 SV = 1	0.02100667	−0.2557645
O02659	Mannose-binding protein C OS=*B. taurus* OX = 9913 GN = MBL PE = 2 SV = 1	0.01287941	−0.2735407
P68401	Platelet-activating factor acetylhydrolase IB subunit alpha2 OS=*B. taurus* OX = 9913 GN=PAFAH1B2P68402 PE = 1 SV = 1	0.02799052	−0.27903
A0AAA9S530	Protein FAM3C OS=*B. taurus* OX = 9913 GN=FAM3C PE = 3 SV = 1	0.02262449	−0.3234315
A0A3Q1MBY4	cAMP-dependent protein kinase type I-alpha regulatory subunit OS=*B. taurus* OX = 9913 GN=PRKAR1A PE = 3 SV = 1	0.02247949	−0.3493251
A5PJB8	CTRB1 protein OS=*B. taurus* OX = 9913 GN=CTRB1 PE = 2 SV = 1	0.02133401	−0.3853862
A0A3Q1MY30	Laminin subunit beta 1 OS=*B. taurus* OX = 9913 GN = LAMB1 PE = 4 SV = 2	0.01481081	−0.4225352
A0A452DJ62	Thrombospondin 4 OS=*B. taurus* OX = 9913 GN = THBS4 PE = 3 SV = 1	0.04360104	−0.4881787
A0A3Q1MT60	Macrophage migration inhibitory factor OS=*B. taurus* OX = 9913 GN = MIF PE = 1 SV = 1	0.03459517	−0.5064463
P19879	Mimecan OS=*B. taurus* OX = 9913 GN=OGN PE = 1 SV = 3	0.00497834	−0.7639411

### CAPG alters the secretome of the endometrium-on-a-chip

**Figure 4:**
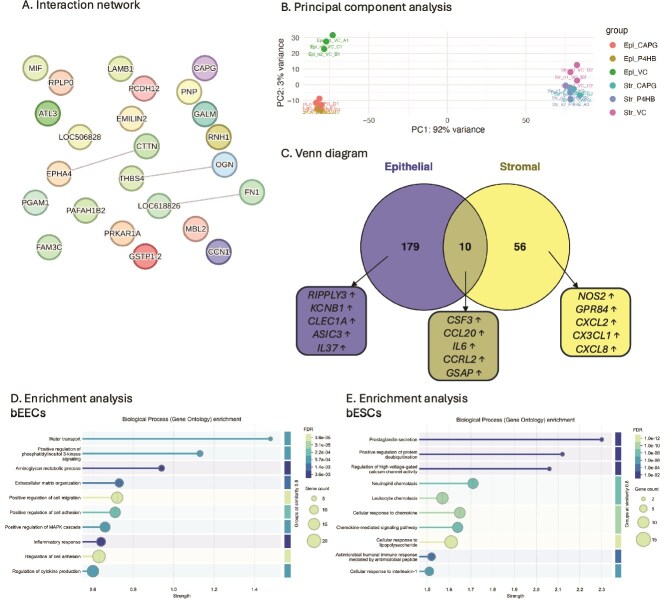
*Impact of CAPG on endometrial secretome and transcriptome. A.* Interaction analysis of differentially abundant proteins in conditioned medium supplemented with rbCAPG compared to vehicle control samples (p < 0.05). Each node represents a protein, nodes with edges represent functional/physical protein associations with a minimum required interaction score of “medium 0.4”. CAPG was added to the culture medium in bEEC culture chamber. Thickness of edge represents the strength of supporting data. Produced using STRING DB. *B.* Principal component analysis of transcriptome of 3D endometrium-on-a-chip epithelial and stromal cells. bEECs and bESCs were cultured in a 3D microfluidic chip, with the bEECs under flow (0.8 μL/min) with culture medium containing rbCAPG (CAPG) or rbPDI (P4HB) 1 μg/mL, or PBS vehicle controls (VC) for 24 h. Epi_ = epithelial cells, Str_ = stromal cells, VC = vehicle control. *C.* Venn diagram of differentially expressed genes in response to rbCAPG treatment. rbCAPG treatment in microfluidic flow through resulted in differentially expressed genes in bovine epithelial and stromal cells when compared to vehicle control samples (n = 3, padj<0.05, fold change <1 or > 1). Produced in Venny. ↑ upregulated, ↓ downregulated. Top 5 up/downregulated genes shown. Full data in Supplementary Table S8. *D.* Biological processes gene ontologies enriched in bovine epithelial cells inside 3D microfluidic organ-on-a-chip device in response to rbCAPG treatment. Analysis carried out in STRING DB, FDR < 0.05, strength of enrichment is Log10(observed/expected). Full data in Supplementary Table S9. *E.* Biological processes gene ontologies enriched in bovine stromal cells inside 3D microfluidic organ-on-a-chip device in response to rbCAPG treatment. Analysis carried out in STRING DB FDR < 0.05, strength of enrichment is Log10(observed/expected). Full data in Supplementary Table S10.

The addition of rbCAPG to the microfluidic flow altered abundance of 24 proteins in the conditioned medium compared to vehicle control samples, including the CAPG protein as the most highly abundant (Table 2). STRING DB analysis of the 24 proteins mapped 20 proteins to the database and revealed that many nodes had no connecting edges, and therefore no known direct interactions, but revealed some interacting clusters, including osteoglycin/mimecan (OGN) and thrombospondin-4 (THBS4), cortactin (CTTN) and receptor protein tyrosine kinase (EPHA4), and fibronectin (FN1) and CTRB1 protein (LOC618826) (Figure 4A).

To determine whether CAPG altered any of the in vitro ULF proteins, a comparison of proteins identified as differentially abundant in the conditioned medium compared to the unconditioned samples (Supplementary Table S2), and proteins identified in the CAPG-treated samples as differentially abundant compared to vehicle control samples (Table 2) was performed. Five proteins were identified as differentially abundant in both: fibronectin (FN1), atlastin GTPase 3 (GNSTP1), glutathione S-transferase P (GSTP1), pregnancy zone protein (LOC506828), and CCN family member 1 (CCN1). ATL3 and FN1 were increased (secreted) in the in vitro ULF and further increased following exposure to rbCAPG (Table 3). CCN1 was increased in the in vitro ULF but decreased marginally by exposure to rbCAPG. Pregnancy zone protein was decreased in the in vitro ULF and further marginally decreased by exposure to rbCAPG, whereas GSTP1 was decreased in the in vitro ULF but increased by rbCAPG (Table 3).

**Table 3:** Proteins altered in conditioned medium and in response to rbCAPG. Proteins identified by TMT mass spectrophotometry in conditioned medium from vehicle control (VC) samples n = 3 significantly differentially abundant (p < 0.05) compared to unconditioned medium samples (n = 2) or conditioned CAPG samples (rbCAPG added to medium, n = 3) compared to conditioned VC samples. Conditioned medium obtained after flowing through a 3D microfluidic chip containing bEECs and bESCs. Medium collected after 24 h from the bEEC compartment. OS = OrganismName, OX = OrganismIdentifier, GN = GeneName, PE = ProteinExistence, SV = SequenceVersion

Accession	Protein	Fold change VC vs unconditioned	Fold change CAPG vs VC
A0A3Q1NHX9	Fibronectin OS=*Bos taurus* OX = 9913 GN=FN1 PE = 4 SV = 1	0.97789813	0.85758831
A0A3Q1LYU3	Atlastin GTPase 3 OS=*B. taurus* OX = 9913 GN = ATL3 PE = 3 SV = 2	3.44215146	0.61717601
P28801	Glutathione S-transferase P OS=*B. taurus* OX = 9913 GN = GSTP1 PE = 1 SV = 2	−1.5079072	0.23242943
F1MJK3	Pregnancy zone protein OS=*B. taurus* OX = 9913 GN = LOC506828 PE = 3 SV = 4	−1.5193085	−0.0578733
Q3ZC35	CCN family member 1 OS=*B. taurus* OX = 9913 GN=CCN1 PE = 2 SV = 1	3.45643383	−0.1192773

### CAPG alters the endometrial transcriptome in vitro

Principal component analysis revealed that the largest contribution to transcriptional difference and clustering between samples was cell type (stromal or epithelial) as expected, with epithelial cell samples clustering separately from stromal cells in PC1 (Figure 4B).

Exposure of epithelial cells to rbCAPG under flow altered expression of 171 protein-coding genes (165 upregulated and 6 downregulated) and 18 long non-coding RNAs (17 upregulated and 1 downregulated) when compared to control (Supplementary Table S6). Similarly, rbCAPG addition altered 61 protein-coding genes (58 upregulated and 3 downregulated) and 5 long non-coding RNAs (all upregulated) in stromal cells when compared to the vehicle control samples (Supplementary Table S7). Ten protein-coding transcripts were commonly altered in both epithelial and stromal cell types (Figure 4C; Supplementary Table S8). All 10 shared transcripts were upregulated compared to vehicle control in both stromal and epithelial cell types.

The 171 protein-coding genes altered by rbCAPG compared to vehicle control in epithelial cells in this 3D microfluidic system were subjected to biological processes GO enrichment analysis in STRING DB, which mapped to 154 proteins. Analysis revealed that water transport, positive regulation of phosphatidylinositol 3-kinase signaling, and aminoglycan metabolic process were the most highly enriched biological processes (Figure 4D: Supplementary Table S9). Enrichment analysis of the 61 protein-coding genes (55 mapped in STRING DB) altered by the addition of rbCAPG when compared to vehicle control samples in stromal cells demonstrated that prostaglandin secretion and positive regulation of protein deubiquitination were the most highly enriched biological process gene ontologies (Figure 4E: Supplementary Table S10).

### PDI alters the secretome of the endometrium-on-a-chip

**Figure 5:**
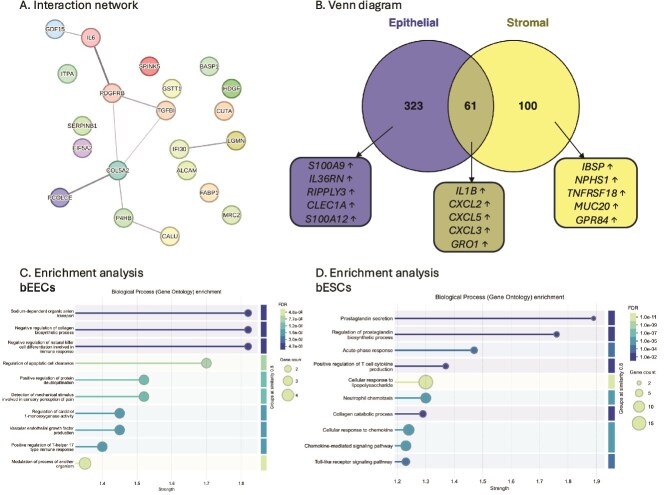
*Impact of PDI on endometrial secretome and transcriptome. A.* Interaction analysis of differentially abundant proteins in conditioned medium supplemented with rbPDI compared to vehicle control samples (p < 0.05). Each node represents a protein, and nodes with edges represent functional/physical protein associations with a minimum required interaction score of “medium 0.4”. PDI (P4HB in figure) was added to the culture medium. Thickness of edge represents the strength of supporting data. Produced using STRING DB. *B.* Venn diagram of significantly altered transcripts in response to rbPDI in different cell types within the bovine endometrium-on-a-chip microfluidic device. Significantly altered transcripts (padj<0.05, >1 or < −1 fold change) enrichment compared to vehicle control samples. ↑ upregulated, ↓ downregulated. Top 5 up/downregulated genes shown. Full data in Supplementary Table S13. *C.* Biological process gene ontologies enriched in bovine epithelial cells inside 3D microfluidic organ-on-a-chip device in response to rbPDI treatment. Analysis carried out in STRING DB FDR < 0.05, strength of enrichment is Log10(observed/expected). Full data in Supplementary Table S14. *D.* Biological process gene ontologies enriched in bovine stromal cells inside 3D microfluidic organ-on-a-chip device in response to rbPDI treatment. Analysis carried out in STRING DB FDR < 0.05, strength of enrichment is Log10(observed/expected). Full data in Supplementary Table S15.

The addition of rbPDI altered abundance of 27 proteins in the conditioned medium compared to vehicle control samples, with PDI the most highly abundant (Table 4). STRING DB analysis of the 27 proteins mapped to 21 proteins in the STRING database, which revealed that many nodes had no connecting edges, and therefore no known interactions, but revealed a clear main interacting clusters, and a small cluster comprised of gamma-interferon-inducible lysosomal thiol reductase (IFI30) and legumain (LGMN) (Figure 5A). The main cluster is centralized around collagen type V alpha 2 chain (COL5A2) as a “hub” protein. Interestingly, PDI (termed P4HB in Figure 5A) was also shown to interact directly with two of the differentially abundant proteins altered in response to PDI exposure—COL5A2 and CALU.

**Table 4:** List of differentially abundant proteins in culture medium following PDI treatment. rbPDI added to the culture medium flowing through the microfluidic bovine endometrium-on-a-chip device for 24 h and proteins present in the conditioned medium determined by mass spectrophotometry, differentially abundant proteins identified compared to vehicle control samples (n = 3 biological replicates). OS = OrganismName, OX = OrganismIdentifier, GN = GeneName, PE = ProteinExistence, SV = SequenceVersion

Accession	Description	*t*-test	fold change
P05307	Protein disulfide-isomerase OS=*Bos taurus* OX = 9913 GN=P4HB PE = 1 SV = 1	0.00042615	4.47464606
P26892	Interleukin-6 OS=*B. taurus* OX = 9913 GN=IL6 PE = 2 SV = 1	0.02343943	2.54836479
Q2HJB6	Procollagen C-endopeptidase enhancer OS=*B. taurus* OX = 9913 GN=PCOLCE PE = 2 SV = 1	0.0296333	0.59034027
A0AAA9SEK8	Semaphorin 7A OS=*B. taurus* OX = 9913 GN=SEMA7A PE = 3 SV = 1	0.04040619	0.58197926
A0A3Q1M0D3	Heparin binding growth factor OS=*B. taurus* OX = 9913 GN=HDGF PE = 1 SV = 1	0.02075538	0.56895933
Q2NL00	Glutathione S-transferase theta-1 OS=*B. taurus* OX = 9913 GN = GSTT1 PE = 2 SV = 3	0.01747217	0.54067143
A0AAA9SNN4	Phosphoglycerate kinase OS=*B. taurus* OX = 9913 GN=PGK1 PE = 1 SV = 1	0.01627738	0.42874025
F1N2Y2	Collagen type V alpha 2 chain OS=*B. taurus* OX = 9913 GN=COL5A2 PE = 4 SV = 4	0.03815541	0.33555225
A0AAA9TXL7	Cadherin 19 OS=*B. taurus* OX = 9913 GN=CDH19 PE = 4 SV = 1	0.00077795	0.31179641
A0A3Q1LN63	Serpin family B member 1 OS=*B. taurus* OX = 9913 GN=SERPINB1 PE = 1 SV = 1	0.02488382	0.27934918
P55906	Transforming growth factor-beta-induced protein ig-h3 OS=*B. taurus* OX = 9913 GN = TGFBI PE = 1 SV = 2	0.01124876	0.20176283
Q95M12	Legumain OS=*B. taurus* OX = 9913 GN = LGMN PE = 1 SV = 1	0.01980579	0.19598183
F1N5T0	CutA divalent cation tolerance homolog OS=*B. taurus* OX = 9913 GN=CUTA PE = 3 SV = 2	0.04723157	−0.0996288
A0AAA9S6S7	Haloacid dehalogenase-like hydrolase domain-containing protein 2 OS=*B. taurus* OX = 9913 GN=HDHD2 PE = 1 SV = 1	0.04214462	−0.1134378
A0A3Q1NA28	CD166 antigen OS=*B. taurus* OX = 9913 GN = ALCAM PE = 4 SV = 2	0.01155258	−0.1839716
P80724	Brain acid soluble protein 1 OS=*B. taurus* OX = 9913 GN=BASP1 PE = 1 SV = 3	0.03060652	−0.1853319
F1N759	Platelet-derived growth factor receptor beta OS=*B. taurus* OX = 9913 GN=PDGFRB PE = 3 SV = 3	0.02438756	−0.1859041
A0A3Q1NC75	Calumenin OS=*B. taurus* OX = 9913 GN=CALU PE = 1 SV = 2	0.04957712	−0.2160784
Q862H7	Protein S100 (Fragment) OS=*B. taurus* OX = 9913 GN=S100A11 PE = 1 SV = 1	0.03834507	−0.2395831
A0A3Q1M5H7	Mannose receptor C type 2 OS=*B. taurus* OX = 9913 GN = MRC2 PE = 4 SV = 2	0.01224295	−0.2772061
A6QPN6	Gamma-interferon-inducible lysosomal thiol reductase OS=*B. taurus* OX = 9913 GN=IFI30 PE = 2 SV = 1	0.04181588	−0.2832836
A0AAA9T1E6	Joining chain of multimeric IgA and IgM OS=*B. taurus* OX = 9913 GN = JCHAIN PE = 1 SV = 1	0.03422581	−0.2915258
Q2KIC5	Inosine triphosphate pyrophosphatase OS=*B. taurus* OX = 9913 GN=ITPA PE = 2 SV = 1	0.02428514	−0.3381766
E1BBL5	Growth differentiation factor 15 OS=*B. taurus* OX = 9913 GN = GDF15 PE = 3 SV = 3	0.04859836	−0.4081335
F1MN49	Eukaryotic translation initiation factor 5A2 OS=*B. taurus* OX = 9913 GN = EIF5A2 PE = 3 SV = 3	0.04615419	−0.5516617
P80425	Fatty acid-binding protein, liver OS=*B. taurus* OX = 9913 GN=FABP1 PE = 1 SV = 1	0.01761834	−0.6901331
F1MJH0	Serine peptidase inhibitor Kazal type 5 OS=*B. taurus* OX = 9913 GN=SPINK5 PE = 4 SV = 4	0.00473122	−0.7125719

To determine whether PDI altered any of the in vitro ULF proteins, a comparison of proteins identified as differentially abundant in the conditioned medium compared to the unconditioned samples (Supplementary Table S2), and proteins identified in the PDI-treated samples as differentially abundant compared to vehicle control samples (Table 4) was performed. Seven proteins were identified as differentially abundant in both analyses (Table 5): interleukin 6 (IL6), procollagen C-endopeptidase enhancer (PCOLCE), legumain (LGMN), cutA divalent cation tolerance homolog (CUTA), protein S100 (S100A11), growth differentiation factor 15 (GDF15), and fatty acid-binding protein (FABP1). IL6 and PCOLCE were secreted in the in vitro ULF and further increased by rbPDI (Table 5). S100A11 and GDF15 were also secreted in the in vitro ULF but decreased by exposure to rbPDI. CUTA, LGMN, and FABP1 were all decreased in the in vitro ULF (taken up from the medium), and CUTA and FABP1 were all marginally further decreased by rbPDI, whereas LGMN was increased by rbPDI (Table 5).

### PDI alters the endometrial transcriptome in vitro

Exposure of epithelial cells to rbPDI altered expression of 358 protein-coding transcripts (325 increased and 33 decreased compared to vehicle control samples) and 26 long non-coding RNA transcripts (23 increased and 3 decreased compared to vehicle control samples) in the epithelial cells (Supplementary Table S11). In stromal cells, the expression of 153 protein-coding transcripts (138 increased and 15 decreased compared to vehicle controls) and 8 long non-coding RNAs (all increased) were significantly altered following rbPDI supplementation to the culture medium (Supplementary Table S12).

Venn diagram analysis demonstrated that 61 of the genes differentially expressed in response to rbPDI exposure were commonly altered in both cell types (Figure 5B: Supplementary Table S13). All commonly altered transcripts were upregulated compared to vehicle control in both epithelial and stromal cell types.

Enrichment analysis identified that the addition of rbPDI altered 384 genes in the epithelial cells (329 mapped in STRING DB), which were significantly enriched in biological processes when compared to vehicle control samples, such as sodium-dependent organic anion transport, negative regulation of collagen biosynthetic process, and negative regulation of natural killer cell differentiation involved in immune process (FDR <0.05, Figure 5C, Supplementary Table S14). Biological process GO terms enriched in the stromal cell compartment (141 of 161 genes mapped in STRING DB) included prostaglandin secretion, regulation of prostaglandin biosynthetic process, and acute-phase response as the most highly enriched (FDR <0.05, Figure 5D, Supplementary Table S15).

**Figure 6:**
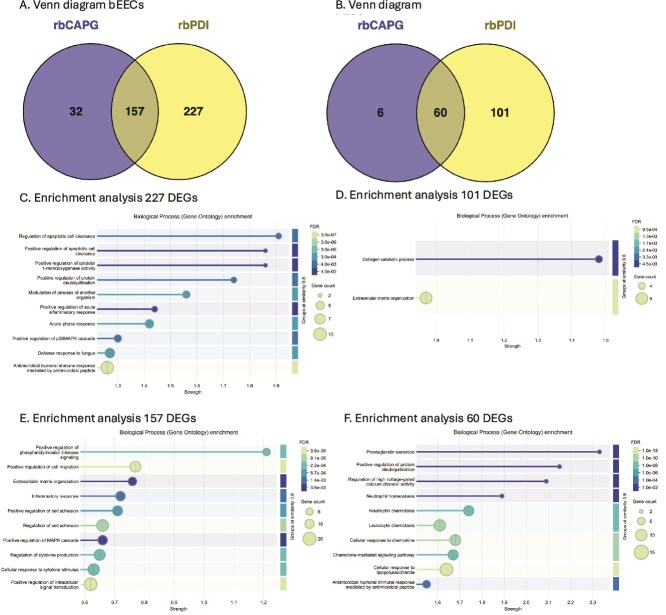
*Response to CAPG and PDI is protein specific.* Venn diagram comparison of rbCAPG and rbPDI induced DEGs in A) bEECs and B) bESCs. Cells treated with 1 μg/mL protein in 0.8 μL/min flow through the bEEC chamber of an endometrium-on-a-chip device also containing bESCs seeded on the other side of a porous membrane. DEGs determined in comparison to vehicle control samples, fold change >1 or < −1 and padj<0.05. Full data in Supplementary Tables S16 and S17. Figures produced in Venny. *C.* Enriched GO terms associated with 177 DEGs specific to rbPDI treatment but not rbCAPG treatment in bEECs. Cells treated with 1 μg/mL proteins under flow and DEGs determined in comparison with vehicle control samples, fold change >1 or < −1 and padj<0.05. Analysis carried out in STRING DB FDR < 0.05, strength of enrichment is Log10(observed/expected). Full data in Supplementary Table S18. *D.* Enriched GO terms associated with 42 DEGs specific to rbPDI treatment but not rbCAPG treatment in bESCs. Cells treated with 1 μg/mL proteins under flow and DEGS determined in comparison to vehicle control samples, fold change >1 or < −1 and padj<0.05. Analysis carried out in STRING DB FDR < 0.05, strength of enrichment is Log10(observed/expected). Full data in Supplementary Table S19. *E.* Enriched GO terms associated with 205 DEGs commonly altered by rbPDI and rbCAPG treatment in bEECs. Cells treated with 1 μg/mL proteins under flow and DEGS determined in comparison with vehicle control samples, fold change >1 or < −1 and padj<0.05. Analysis carried out in STRING DB FDR < 0.05, strength of enrichment is Log10(observed/expected). Full data in Supplementary Table S20. *F.* Enriched GO terms associated with 37 DEGs commonly altered by rbPDI and rbCAPG treatment in bESCs. Cells treated with 1 μg/mL proteins under flow and DEGS determined in comparison to vehicle control samples, fold change >1 or < −1 and padj<0.05. Analysis carried out in STRING DB FDR < 0.05, strength of enrichment is Log10(observed/expected). Full data in Supplementary Table S21.

To understand if there was a protein specific response in the different cell types, we compared DEGs in response to CAPG and PDI exposed bEECs (Figure 6A) and bESCs (Figure 6B). One hundred and fifty-seven transcripts were commonly altered following rbCAPG and rbPDI treatment, whereas 32 transcripts were specifically in response to rbCAPG and 227 transcripts specific to rbPDI epithelial cells (Figure 6A, Supplementary Table S16). In bESCs, 60 transcripts were commonly altered in response to rbCAPG and rbPDI treatment, whereas six transcripts were altered specifically in response to rbCAPG and 101 transcripts were altered specifically in response to rbPDI (Figure 6B, Supplementary Table S17).

Enrichment analysis on the 32 transcripts specific to rbCAPG in bEECs or the six transcripts specific to rbCAPG in bESCs did not reveal any significantly enriched GO terms. The 227 transcripts specific to rbPDI in bEECs revealed enrichment in 204 GO terms, including regulation of apoptotic cell clearance, positive regulation of apoptotic cell clearance, and positive regulation of calcidiol 1-monooxygenase activity (Figure 6C, Supplementary Table S18). Enrichment analysis on the 101 transcripts specific to rbPDI treatment in bESCs revealed enrichment in two GO terms: collagen catabolic process and extracellular matrix organization (Figure 6D, Supplementary Table S19). Enrichment analysis on the 157 DEGs commonly elicited by both rbCAPG and rbPDI in bEECs identified 32 enriched GO terms, including positive regulation of phosphatidylinositol 3-kinase signaling, positive regulation of cell migration, and extracellular matrix organization (Figure 6E, Supplementary Table S20). Similarly, analysis on the 60 DEGs commonly altered by both rbCAPG and rbPDI in bESCs identified 80 enriched GO terms, including prostaglandin secretion, positive regulation of protein deubiquitination, and regulation of high voltage-gated calcium channel activity (Figure 6F, Supplementary Table S21).

### Impact of culture model on endometrial response to conceptus-derived proteins

**Figure 7:**
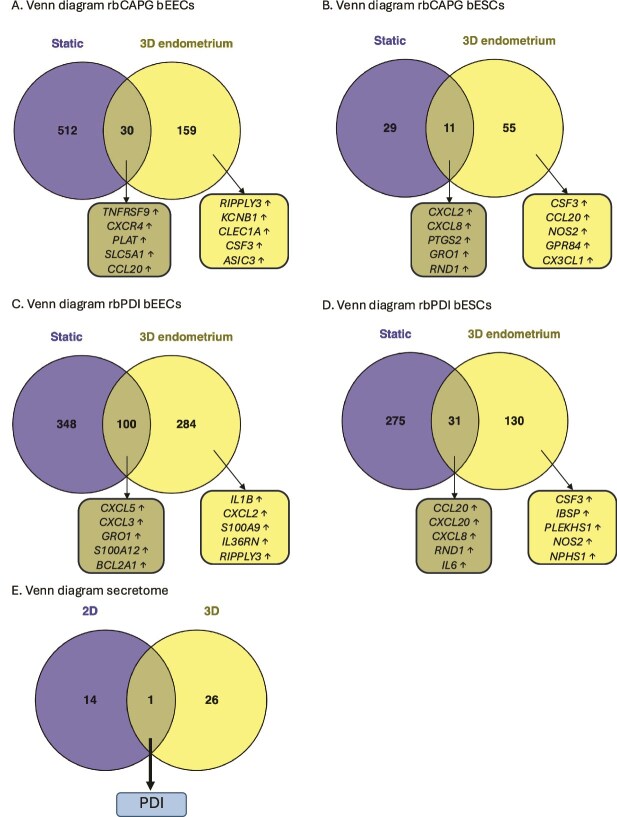
*Comparison of differentially expressed genes or altered secretome from 2D-static vs 3D-endometrium-on-a-chip systems treated with rbCAPG and PDI.* Static culture system was 2D and a single monolayer of cells presented in Chapter 2. 3D endometrium-on-a-chip system is a 3D culture of both epithelial and stromal cells, with rbPDI or rbCAPG applied to the epithelial cell side under flow 0.8 μL/min. Differentially expressed genes (padj <0.05, fold change >1 or < −1) determined following rbCAPG treatment in *A.* bEECs or *B.* bESCs compared to vehicle control samples, or following rbPDI treatment in *C.* bEECs or *D.* bESCs compared to vehicle control samples. ↑ upregulated, ↓ downregulated. Top 5 up/downregulated genes shown. Full data in Supplementary Tables S22–S25. *E.* Comparison of the conditioned medium and secretome of 2D and 3D microfluidic culture systems following rbPDI treatment. 2D culture system from Tinning et al. [30] consisted of bovine endometrial epithelial cells in a simple microfluidic channel in a monolayer. The 3D microfluidic culture system presented here contained both stromal and epithelial cells applied to either side of a porous membrane. Proteins were identified by TMT mass spectrophotometry compared to vehicle control samples (p < 0.05). Full data in Supplementary Table S26.

To understand how the 3D endometrium-on-a-chip culture system differs in responding to conceptus-derived proteins, the transcriptional data from the static 2D cell culture transcriptional response to rbCAPG [28] and rbPDI [30] were compared to data produced in this experiment. When epithelial cells were exposed to rbCAPG, 30 DEGs were commonly altered in both the 2D-static and 3D-flow systems, with 159 DEGs specific to the 3D-flow endometrium-on-a-chip system and 512 DEGs specific to 2D culture (Figure 7A: Supplementary Table S22). Second, the stromal cell transcriptional response from both systems was compared, revealing only 11 shared DEGs between both systems, with 55 DEGs specific to the 3D endometrium-on-a-chip system and 29 DEGs specific to the static culture system in response to rbCAPG (Figure 7B: Supplementary Table S23).

In epithelial cells exposed to rbPDI, 100 DEGs were commonly altered between 2D-static and 3D-Flow systems, with a further 284 DEGs specific to the 3D-flow and 348 DEGs specific to 2D-static culture (Figure 7C: Supplementary Table S24). Second, the stromal cell transcriptional response from both systems was compared, revealing only 31 shared DEGs between both systems, with 130 DEGs specific to the 3D endometrium-on-a-chip system and 275 DEGs specific to 2D static culture (Figure 7D: Supplementary Table S25). Finally, the secretome of the cells cultured within the 3D endometrium-on-a-chip in response to rbPDI (Table 5) was compared to the secretome of epithelial cells cultured in a 2D microfluidic system in response to rbPDI [30]. No commonly altered proteins were identified (aside from the rbPDI added to the culture medium), 26 proteins were specifically altered in the 3D endometrium-on-a-chip system, and 14 proteins were specifically altered in static 2D culture (Figure 7E: Supplementary Table S26).

**Table 5:** Proteins altered in conditioned medium and in response to rbPDI. Proteins identified by TMT mass spectrophotometry in conditioned medium from vehicle control (VC) samples n = 3 significantly differentially abundant (p < 0.05) compared to unconditioned medium samples (n = 2) or conditioned PDI samples (rbPDI added to medium, n = 3) compared to conditioned VC samples. Conditioned medium obtained after flowing through a 3D microfluidic chip containing bEECs and bESCs. Medium collected after 24 h from the bEEC compartment. OS = OrganismName, OX = OrganismIdentifier, GN = GeneName, PE = ProteinExistence, SV = SequenceVersion

Accession	Protein	Fold change VC vs unconditioned (in vitro ULF)	Fold change PDI vs VC
P26892	Interleukin-6 OS=*Bos taurus* OX = 9913 GN=IL6 PE = 2 SV = 1	3.39997164	2.54836479
Q2HJB6	Procollagen C-endopeptidase enhancer OS=*B. taurus* OX = 9913 GN=PCOLCE PE = 2 SV = 1	1.29114066	0.59034027
Q95M12	Legumain OS=*B. taurus* OX = 9913 GN = LGMN PE = 1 SV = 1	−1.4108192	0.19598183
F1N5T0	CutA divalent cation tolerance homolog OS=*B. taurus* OX = 9913 GN=CUTA PE = 3 SV = 2	−0.7187638	−0.0996288
Q862H7	Protein S100 (Fragment) OS=*B. taurus* OX = 9913 GN=S100A11 PE = 1 SV = 1	1.40124894	−0.2395831
E1BBL5	Growth differentiation factor 15 OS=*B. taurus* OX = 9913 GN = GDF15 PE = 3 SV = 3	1.38562079	−0.4081335
P80425	Fatty acid-binding protein, liver OS=*B. taurus* OX = 9913 GN=FABP1 PE = 1 SV = 1	−1.558239	−0.6901331

## Discussion

To understand how conceptus-derived proteins may alter the transcriptome and secretome of the endometrium in vivo*,* we used a 3D bovine multicellular endometrium-on-a-chip device in vitro to aid in deciphering the complex crosstalk between endometrium and conceptus. We determined how the “in vitro” ULF secretome differed from the in vivo ULF proteins identified [5], and the transcriptional and secretory response to conceptus-derived proteins (CAPG and PDI). Finally, we determined how this differs from traditional static 2D in vitro culture systems. This 3D endometrial model has two chambers separated by a porous membrane, allowing cellular communication through the membrane similar to endometrial and stromal crosstalk that occurs in vivo. The static chamber represents the underlying stromal compartment, and the microfluidic channel under flow represents the uterine lumen and epithelial monolayer lining the endometrial tissue. This design was used to recapitulate the communication that occurs between conceptus and endometrium in vivo. The bovine conceptus elongates to fill the ipsilateral horn by day 16, both horns by day 21[48, 49], with the outermost trophectoderm cells of the conceptus closely aligned with the luminal epithelium. Therefore, the luminal epithelial cells would be exposed to any conceptus-derived secretions first. Using this orientation (Figure 1), the conceptus-derived proteins could be applied under gentle flow, to allow the endometrial response to conceptus-derived proteins, while collection of the conditioned medium also gave an insight into the secretome of the epithelial cells within the system (representing the ULF in vitro).

### ULF proteins secreted by the 3D in vitro endometrium may be involved in adhesion and supporting endometrial development in vivo

Enrichment analysis revealed proteins associated with the GO terms calcium-independent cell-matrix adhesion, actin cytoskeleton reorganization, and intermediate filament cytoskeleton organization. This indicates that many of the proteins secreted into the in vitro ULF may be involved in the establishment of the endometrial tissue within the system. In our system, it took 3–4 days to develop to the epithelium to point of 90% confluence and was left intact for another 24 h. This is a relatively short time-frame and likely resulted in tissue-like compartments still forming an intact monolayer of epithelial cells (forming cell–cell adhesions), and attaching to the underlying substrate (the membrane and/or ibiTreat Ibidi polymer material) (Xavier [50]). Other studies attempting to recapitulate the 3D bovine endometrium in vitro which have assessed tissue structure used culture lengths of 14 [17] and 35 days [51]. Further optimizing the culture medium may produce a secretome, which further matches that of the in vivo produced ULF.

### In vitro bovine ULF has limited similarity to in vivo ULF

Proteomic analysis of the spent conditioned culture medium flowed through the microfluidic channel revealed that 69 proteins were actively secreted (mimicking the ULF secretion). When compared to a published dataset of day 16 non-pregnant bovine ULF proteome [5], only six proteins were shared. Given that only half of the proteins identified in the study by Forde et al. were successfully converted to the same protein identifier used here, it is possible that a greater number of proteins were shared with the proteins secreted in this in vitro system but were lost in the data processing steps.

Of the six identified proteins, both in vitro in the 3D system and in in vivo ULF, keratin 19 has previously been identified as being increased in abundance in the ULF from day 8 pregnant cattle compared to cyclic cattle [52]. Its expression in bovine embryos is higher in in vitro fertilized than in lower competency nuclear transfer embryos [53]. Keratin 19 may therefore be secreted to support conceptus development in cases of lower competency. Annexin A2 is involved in attachment of murine blastocysts in vitro*,* and infusion of annexin A2 artificially into the murine uterus increased the number of implantation sites [54]. Annexin A2 has also been shown to be upregulated during the estrus phase (cycle day 20–22 postovulation) compared to the diestrus phase (cycle days 5–15 postovulation) in cattle, which the authors attribute to rising P4 concentration in the maternal circulation acting upon the endometrium [55]. Conversely, in humans, annexin A2 was found to be more highly expressed in the endometrium during the midsecretory phase (approximately cycle day 21 postmenses start, and approximately day 7 postovulation) compared to the late proliferative phase (cycle days 8–10 postmenses start) [56]. The midsecretory phase corresponds to the stage at which the human embryo implants (days 6–9 postovulation). This may point to an important role in the endometrial production of annexin A2 in supporting implantation, in a species-specific manner.

The protein basal cell adhesion molecule is an immunoglobulin adhesion molecule involved in cell–cell adhesion [57]. It is also highly differentially expressed in the endometrium compared to the conceptus in ovine and interacts with secretory protein LAMA5 [58]. Although little is currently known about its role in implantation, it could be involved in cell–cell adhesion of the conceptus and endometrium during implantation. Although the 3D endometrial model presented here does not fully recapitulate the in vivo secreted ULF. The composition of ULF in vivo varies throughout the estrus cycle [59–62] and by pregnancy stage ([63, 64]; [65]); therefore, the ULF is variable dependent upon exposure to cycle-related changes in maternal hormones and the influence of the conceptus. The 3D endometrium-on-a-chip system presented here could be adapted to investigate the influence of steroid hormone exposure (by adding the upper stromal “maternal-side” chamber) or conceptus-derived factors (by adding to the lower epithelial “conceptus-side” channel. The device can also be used in reverse, with the “maternal-side” stromal cells in the lower flow channel to recapitulate the maternal circulatory system, and has been used to investigate the influence of maternal glucose and insulin concentrations [27], which further influences the secretome in the system.

### CAPG alters the secretome and proteome of the 3D in vitro endometrium-on-a-chip

When rbCAPG was added to the microfluidic flow through on the endometrial epithelial side (replicating the uterine luminal side), it was to replicate how CAPG would be secreted from the conceptus and how the endometrium would be exposed to CAPG in vivo*.* This resulted in a change in the secretome of the in vitro ULF produced. One of the altered in vitro ULF proteins, macrophage inhibitory factor (MIF), was previously identified in the bovine [5] pregnant ULF as a “non-classical ISG protein” (i.e., not associated with a type I interferon response), secreted by bovine epithelial cells in vitro in response to IFNT [66], and shown to be secreted by human endometrial cells in response to hCG [67]. MIF’s main function is as a pro-inflammatory cytokine and has been discussed to have potential roles in many areas of reproductive biology [68]. Interestingly, MIF has been shown to stimulate migration and invasion of trophoblast cell lines in vitro [69] as well as many roles in supporting placental development and function [68]. Therefore, CAPG may be secreted by the conceptus to stimulate the endometrium to secrete MIF, alongside IFNT (or hCG in humans), to support implantation and placentation.

Two other proteins, which were secreted in response to CAPG which are predicted to interact—OGN and THBS4. OGN is a growth factor expressed only in the endometrium and not in the bovine conceptus, and THBS4 is expressed as a ligand on the bovine conceptus [70]. CAPG may activate secretion of these proteins to further promote physical interactions between the conceptus and the endometrium and to stimulate conceptus growth during elongation.

A comparison of the proteins differentially abundant in vitro within the 3D endometrium on a chip system (in vitro ULF proteins) to those altered following exposure to rbCAPG revealed five overlapping proteins—specifically, atlastin GTPase 3 (ATL3) was increased in abundance in the in vitro ULF and further increased following rbCAPG exposure. ATL3 is a receptor GTPase involved in the endoplasmic reticulum, promoting tubular fusion and degradation of the tubular endoplasmic reticulum [71]. ATL3 has not yet been discussed in the literature in the context of ULF.

### CAPG may alter the endometrial transcriptome to mediate the immune response and remodel the endometrium

Treatment with rbCAPG increased expression of *MMP13* to the greatest extent. A matrix metalloproteinase involved in degrading collagenous extracellular matrix, *MMP13* expression, is modulated at the site of implantation in pigs [72], and extracellular matrix remodeling is highly involved in implantation and placentome formation in cattle [73]. *CCL2* was also increased in response to rbCAPG specifically in stromal cells and has previously been shown to be highly expressed in pregnant endometrium in cattle compared to non-pregnant controls on days 15 and 18 of pregnancy. This in vivo expression was not in response to IFNT, as demonstrated by an ex vivo explant culture [74]. Other C-X-C motif chemokines were highly expressed in both cell types in response to rbCAPG. Chemokines have been demonstrated to regulate endometrial–conceptus interactions in cattle [74], pigs [75], and humans [76]. *TNFSF15* was specifically upregulated in stromal cells only, but has previously been identified as a conceptus-specific cytokine [70].

In the endometrial epithelial cells, the biological processes most enriched among genes differentially expressed in response to rbCAPG compared to vehicle control samples included: positive regulation of cell migration, positive regulation of cell adhesion, inflammatory response, and extracellular matrix organization. Cell adhesion is a process required for the attachment of the conceptus trophectoderm to the endometrial epithelium (implantation) [77]. Cell migration and extracellular matrix organization may be related to the epithelial-stromal cell communication to promote endometrial remodeling during early pregnancy. Overall, this study supports the notion that CAPG, secreted by the conceptus, supports immune regulation, modulates secretion of ULF, promotes epithelial–stromal cell communication, and implantation processes in cattle.

### PDI exposure may modify the endometrial transcriptome to mediate the inflammatory response to conceptus

Exposure to rbPDI altered the transcriptome of both epithelial and stromal but to a greater extent in epithelial cells. Some of the most highly induced transcripts in epithelial cells (e.g., *M-SAA3.2, SAA3, S100A8, CLEC7A,* and *S100A9*) are linked to immune response [78–80]. PDI also induced C-X-C motif chemokine expression in both epithelial and stromal cells, similarly to what was observed for rbCAPG. Specifically, in stromal cells, the expression of *CALCB*, involved in placental development, was increased in response to rbPDI. *CALCB* has been identified as expressed during the “window of receptivity” in human endometrium [81].

Many biological processes were enriched among the genes differentially expressed in response to rbPDI, including many involved in immune response, transport, and adhesion in epithelial cells. Similarly, immune response and adhesion-related biological processes were enriched in stromal cells, in addition to prostaglandin secretion and biosynthesis. PDI, therefore, may modulate the immune response to conceptus in vivo and regulate prostaglandin secretion.

### PDI also modifies the endometrial secretome in vitro

Two proteins found to be secreted in response to rbPDI were also shown to directly interact with PDI by interaction analysis- COL5A2 and CALU. COL5A2 has been identified as an endometrial receptivity gene upregulated in response to the embryo in humans [82] and during implantation in rabbits [83]. COL5A2 is a fibrillar type of collagen associated with ECM organization; therefore, PDI may promote COL5A2 secretion to act in an autocrine manner upon the endometrium to support implantation. LGMN was secreted in response to rbPDI and has previously been identified as being upregulated in the bovine endometrium during early pregnancy and speculated to be involved in placentome formation, as it is a protease activator and could therefore be involved in endometrial remodeling [84].

A comparison of the proteins differentially abundant in vitro within the 3D endometrium-on-a-chip system to those altered following exposure to rbPDI revealed seven overlapping proteins. Two of those proteins (LGMN and FABP1) were decreased in the in vitro ULF, indicating that the cells were taking up the proteins from the culture medium rather than secreting them as ULF proteins. IL6 was increased in the in vitro ULF proteins compared to unconditioned samples and further increased by exposure to rbPDI. IL6 has been shown to be increased in the uterine lumen in mice during pregnancy [85] and secreted into the uterine lumen in cattle [86]. IL6 is involved in conceptus development and attachment in pigs [87] and is known to be secreted in response to bacterial infection [88]. Growth differentiation factor (GDF15) was significantly increased in the in vitro ULF but decreased by exposure to rbPDI, indicating that rbPDI decreases secretion of GDF15. GDF15 is involved in promoting embryo endometrial invasion [89] and has recently been linked to causing hyperemesis gravidarum in humans [90]. PDI may therefore alter the endometrial secretome to modulate embryo invasion and mediate the endometrial immune response to conceptus during early pregnancy.

### PDI and CAPG differentially altered the transcriptome in the endometrium-on-a-chip system

A comparison of the transcriptomic response of the epithelial and stromal cells cultured within the 3D endometrium-on-a-chip system revealed that many (37–90% of total DEGs) of the differentially expressed genes were commonly altered between both rbCAPG and rbPDI exposure. This indicates that the proteins may have overlapping functions in vivo. rbCAPG altered minimal (32 transcripts in bEECs and six transcripts in bESCs) DEGs, which were also not altered by rbPDI. Comparing previously published data also demonstrates an overlap in the transcriptional response to rbCAPG [28] and rbPDI [30] under static culture conditions. Enrichment analysis did not reveal any enriched GO terms among the limited DEGs. However, rbPDI altered more transcripts, which were not altered by rbCAPG (227 in bEECs and 101 in bESCs). Enrichment analysis on those 227 DEGs revealed many enriched GO terms, many of which were related to immune system regulation in bEECs, but also apoptotic clearance and protein deubiquitination. The 42 DEGs specific to rbPDI exposure in bESCs identified the GO terms collagen catabolic process and extracellular matrix organization. Remodeling of the endometrium occurs during embryo implantation, and collagens and other extracellular matrix proteins have been shown to be altered during the implantation and placentome formation stages in cattle [73]. Therefore, PDI may have a role in endometrial remodeling during early pregnancy in cattle and be secreted by the conceptus to promote this in vivo*.*

### 3D endometrium-on-a-chip system demonstrates both differences and similarities in secretome and transcriptome response to conceptus-derived proteins compared to static in vitro cell culture

Of the differentially expressed genes elicited by CAPG/PDI in the 3D endometrium-on-a-chip system, between 72 and 95% (varied between cell types and treatment) of those were specific to the 3D culture system, whereas 5–28% of transcripts were also altered in the static 2D systems [28, 30]. This indicates that the endometrial response to conceptus-derived proteins differs when under flow and when epithelial cells are exposed to stromal cells (recapitulating the in vivo endometrial structure), compared to under standard static culture techniques. This reinforces our need to develop 3D in vitro models to study endometrial–conceptus communication to better model the in vivo environment. One gene found highly expressed in response to rbCAPG in the 3D culture system only was *RIPPLY3*, which has previously been found to be upregulated in the endometrium during the window of implantation in pregnant mice [91].

The secretome of the 3D endometrium-on-a-chip from the epithelial side was wholly different from that of the 2D epithelial-only channel in response to rbPDI [30]. This demonstrates that the 3D co-culture of stromal and endometrial cells changes how they function, as evidenced by work showing stromal cells support epithelial cell growth and differentiation in human in vitro cultures [92], further supporting our need for in vitro culture systems which consider and recapitulate aspects of the in vivo environment.

## Conclusion

Future work should further develop in vitro co-culture techniques to investigate early pregnancy events, such as the endometrial response to IFNT (the pregnancy recognition signal in ruminants such as cattle), which has been investigated extensively (reviewed by [93]). The effect of IFNT upon the endometrium has not been investigated in vitro using a microfluidic or organ-on-a-chip approach to date, and therefore would be a logical next step to utilize this technology. Similarly, fabricating devices in which the cells are accessible once seeded would allow investigators to assess the integrity of the epithelial monolayer [94] and incorporating the use of hydrogels or extracellular matrix proteins [95] to culture stromal cells in a 3D conformation rather than a monolayer could further improve the similarity of this in vitro endometrium-on-a-chip to in vivo endometrial tissue. rbPDI and rbCAPG were used at a concentration of 1 μg/mL due to previous work within the group and to be able to directly compare different cell culture systems [28, 30]; however, it is currently unknown what the physiological concentrations of proteins within ULF are. Further work is therefore needed in this area to clarify in vivo concentrations.

In conclusion, we have demonstrated that a 3D bovine microfluidic endometrium-on-a-chip was successfully utilized to mimic the endometrium, ULF secretion, and exposure to conceptus-derived proteins. The in vitro ULF produced from the endometrial side of the 3D bovine endometrium-on-a-chip system had limited similarity to in vivo day 16 non-pregnant ULF, but exposure to conceptus-derived protein, PDI and CAPG altered the secretome and transcriptome of the bovine endometrium-on-a-chip in a protein-specific manner. The endometrial response to rbPDI was also shown to differ in the 3D system compared to a previously used 2D microfluidic system, indicating the importance of using co-culture systems.

## Supplementary Material

Updated_supplementary_tables_ioaf077

## Data Availability

The data discussed in this publication have been deposited in NCBI’s Gene Expression Omnibus [39] and are accessible through GEO Series accession number GSE287563. The mass spectrometry proteomics data have been deposited to the ProteomeXchange Consortium via the PRIDE [40] partner repository with the dataset identifier PXD059725.
